# Early Screening of the Autism Spectrum Disorders: Validity Properties and Cross-Cultural Generalizability of the First Year Inventory in Italy

**DOI:** 10.3390/brainsci10020108

**Published:** 2020-02-18

**Authors:** Annalisa Levante, Serena Petrocchi, Angelo Massagli, Maria Rosaria Filograna, Serafino De Giorgi, Flavia Lecciso

**Affiliations:** 1 Department of History, Society and Human Studies, Department of History, Society and Human Studies, University of Salento, Via di Valesio, 73100 Lecce, Italy; flavia.lecciso@unisalento.it; 2 Lab of Applied Psychology and Intervention, Department of History, Society and Human Studies, University of Salento, Via di Valesio, 73100 Lecce, Italy; serena.petrocchi@usi.ch; 3 Institute of Communication and Health, Università della Svizzera Italiana, Via Buffi 13, 6900 Lugano, Switzerland; 4 Applied Research Division for Cognitive and Psychological Science, IRCCS European Institute of Oncology, Via Ripamonti 435, 20141 Milano, Italy; 5 Child and Adolescents Neuropsychiatry Unit, Local Health Service, Via Miglietta, 73100 Lecce, Italy; angelo.massagli@gmail.com; 6 Federation of Italian Medical Pediatricians, Via Miglietta, 73100 Lecce, Italy; 7 Department of Mental Health, Local Health Service, Via Miglietta, 73100 Lecce, Italy; direzione.dsm@ausl.le.it

**Keywords:** First Year Inventory, autism spectrum disorders, early screening, risk, cross-cultural generalisability, validity

## Abstract

This study examined the cross-cultural generalisability of the First Year Inventory (FYI) on an Italian sample, testing its construct validity, consistency, and structural validity. Six hundred ninety-eight parents of children aged 11–13 months completed the questionnaire. Similarities between analyses of Italian and American/Israeli samples were found, as were demonstrations of the instrument’s construct validity and internal consistency with both groups. The original factorial structure was not demonstrated; thus, a new factorial structure was tested, and a short version of the FYI was demonstrated via confirmatory factor analysis. The findings supported the generalisability of the Italian version of the FYI and its validity. The FYI may aid in medical decision-making on further steps for referral of the child to an early diagnostic assessment.

## 1. Introduction

Autism spectrum disorder (ASD) is a neurodevelopmental condition characterised by (a) persistent deficits in social communication and interaction and (b) restricted and repetitive patterns of behaviours, interests, and/or activities [1]. Recent epidemiological data [2] suggested that the prevalence of ASD reaches the proportion of 1/59 at age 4. To promote early detection of the risk of ASD, as recommended also by the American Academy of Pediatrics [3], several researchers [4,5,6,7,8] developed ad hoc measures for children under 24 months of age that are able to identify behaviours deviating from typical development.

In this vein, a recent systematic review [9] identified 16 Level 1 and 2 screening measures for the early detection of signs of ASD: 4 observational checklists, 2 interviews, and 10 questionnaires. Level 1 screening tools have been developed for the general population to detect children at risk of developmental disorders, including ASD. Level 2 screening measures have been developed to detect children who are at risk for ASD, since they are already referred to the health service for developmental concerns (i.e., low-risk children) or because they are siblings of children with ASD (i.e., high-risk children). This review identified five promising instruments: the First Year Inventory (FYI) [8], the Modified-CHecklist for Autism in Toddler and its revised/follow-up form (M-CHAT and M-CHAT-R/F) [6,10], the Parental Observation of Early Markers Scale (POEMS) [11], and the Quantitative-CHecklist for Autism in Toddler (Q-CHAT) [4]. Analyses of the psychometric properties of these measures evaluated them as good. At the same time, however, the authors stressed that, for several such measures, further validation studies were needed to evaluate certain methodological properties that, as yet, were not adequately investigated.

The highest number of validation studies retrieved in the literature were for the M-CHAT and the M-CHAT-R/F [6,10,12,13,14,15,16,17,18,19,20,21,22,23,24,25,26]. The Q-CHAT has been validated by five studies [4,27,28,29,30] and the FYI by five studies [8,31,32,33,34]. The POEMS has been validated by one study [11].

The M-CHAT and the M-CHAT-R/F can be administered from 16 months of life, the POEMS from 1–24 months of life, the Q-CHAT is administrable when the child is 18 months old, and the FYI when he/she is 11–13 months old. The POEMS requires more administration time as it uses multiple parental observations. The present study focused on the FYI since it allows the earliest screening but—in contrast to the POEMS—requires less administration time and can be completed by parents during regular well-child visits as part of pediatric surveillance.

### The First Year Inventory: Measure Description and Critical Analysis of the Validation Studies

The FYI is a Level 1 screening measure designed to detect the ASD risk on the general population. It was developed through a systematic review of the literature conducted by Reznick and colleagues [8], who identified a list of behaviours comprised in the two core diagnostic criteria of the ASD (i.e., socio-communication and social interaction deficit and restricted, repetitive patterns of behaviour) [1]. Specifically, the authors analysed several retrospective studies and descriptive reports provided by parents, which assessed the first months of life of children with a later diagnosis of ASD, and prospective studies on children who had an older sibling with a diagnosis of ASD. As the authors highlighted, two sets of behaviours, clustered in two categories labelled ‘Social–Communication’ and ‘Sensory–Regulatory Functions’, detect children who are at risk of developing, at an early age, an ASD [8]. The Social–Communication domain was further differentiated into four constructs (Social Orienting & Receptive Communication, Social-Affective Engagement, Imitation, and Expressive Communication) as well as the Sensory–Regulatory Functions domain (Sensory Processing, Regulatory Patterns, Reactivity, and Repetitive Behaviors). For a detailed description of the domains and constructs, please refer to the Appendix of Reznick and colleagues’ paper [8].

The 63 items of the FYI include 46 questions with response options on a four-point Likert scale (from 1—‘never’—to 4—‘often’) and 14 items with answers in a three or four ad hoc multiple-choice format (see Appendix in [8]). Three additional open-ended questions were on (a) the number of consonants used by the child (Item 61); (b) parental concerns or interests about the child’s development (Item 62); and (c) the presence of a specific medical condition (Item 63). Item 61 is scored from 0 (i.e., if the child uses more than three consonants) to 2 points (if the child uses only one or any consonants). The two last open-ended questions (Items 62 and 63) did not receive a score because they were used for qualitative evaluation.

This first study of the FYI was on an American sample (N = 1300) selected from the general population [8], with the purposes of (a) defining the scoring procedure; (b) identifying the risk cut-offs; and (c) evaluating the factorial structure of the instrument. With regard to the scoring procedure, according to the response distribution of the sample, the authors assigned 0 or 1 point to the answers corresponding to behaviours with the highest frequency expected in typically developing children (i.e., low risk). For example, Item 1 (‘Does he/she look at you when his/her name is called?’) received 0 or 1 point when the answer is respectively ‘always’ or ‘sometimes’ because it is expected that a typically developing child looks at the person who calls his/her name. Two points are assigned to answers that have either low frequency (<5%) or correspond to behaviours unusual in typically developing children. For Item 1, the answers ‘never’ and ‘seldom’ receive 2 points because they represent unusual behaviours for a typically developing child.

To identify the cutoffs of risk, the authors [8] observed that the distribution had a chi-squared shape and identified a significant shape inflection corresponding to the score of 17 (at the 95th percentile of the distribution). Finally, they conducted an exploratory factor analysis (EFA), applying the principal factor method followed by a promax (oblique) rotation. The EFA accounted for six factors corresponding to four constructs of the Socio-Communication domain (Social-Affective Engagement—six items, Imitation—four items, Social Orientation—two items, and Expressive Communication—two items) and two constructs of the Sensory–Regulatory Functions domain (Regulatory Patterns—four items and Repetitive Behaviors—eight items). Thirty items did not load for any factor or loaded for more than one factor. To accomplish the broader goal of developing a measure for the early detection of ASD, the authors sorted the 61 items into the hypothesised eight constructs and two domains according to the theoretical model. After the EFA, each of the nonassigned items was allocated to a construct if the item theoretically fitted with that construct, the item–total correlation was higher than 0.30, and the change to Cronbach’s alpha was negligible. After that procedure, nine items were assigned to an uncategorised group because they did not fit any of these criteria.

The FYI was tested in four other studies [31,32,33,34]. One [33] was a follow-up investigation of the Reznick and colleagues’ sample [8] developed three years later. Two were retrospective studies on an American [34] and an Italian sample [32] of children with ASD. Finally, a more recent study [31] was published using an Israeli sample from the general population.

In the following section, we reported a critical comparison between validation studies.

All validation studies carried out analysis on children’s (gender and family size) and parents’ (educational level, ethnicity, and marital status) socio-demographic variables. With regard to the children’s gender, all validation studies found a similar result: all males reached a higher score than females, both in the general [8,32] and clinical [35] population. Only Reznick and colleagues [8] found no significant impact of the family size variable on FYI score. With regard to the parental variables, only two studies [8,32] evaluated them. Specifically, both Reznick and colleagues [8] and Ben-Sasson and [32] found a negative and significant impact of low maternal educational level on FYI score. For this reason, both validation studies suggested rewriting several items. Furthermore, the study by Reznick and colleagues [8] found a significant and positive impact on FYI score for black mothers, whereas Ben-Sasson and Carter [32] found a significant and positive impact of single status mothers on screening measure score. As suggested by these authors [8,32], these variables could be monitored by researchers and professionals to interpret the FYI score adequately.

With regard to the questionnaire psychometric properties, it was worth noting possible detected similarities and differences between the validation studies. The convergent validity was demonstrated by two studies [32,34]. The first study [34] carried the analysis on a sample of the general population recruited by Reznick’ study [8]; the second study [32] analyzed a sample of the Israeli general population. Both validation studies administered the observation and standardized measures to assess the child’ autistic traits (ADOS 2—Autism Diagnostic Observation Schedule-Second Edition—and AOSI—Autism Observation Scale for Infants [36]—respectively) and his/her global functioning (MSEL). Furthermore, both validation studies suggested developing a short version of the FYI. Only Turner-Brown and colleagues [34] examined the accuracy of the screening measure applying a Receiver Operating Characteristic (ROC) analysis: they stated that the combined score on Social–Communicative and Sensory–Regulatory Functions domains was the optimal threshold to detect child at risk at 12 months. In addition, Muratori and colleagues [33] evaluated the FYI accuracy on a clinical sample, and they stated that a two-domains approach of social-communicative and total domains was the optimal threshold to detect cases of early-onset autism. Finally, only Reznick and colleagues [8] demonstrated the questionnaire structural validity and carried out an Explorative Factor Analysis (EFA). As anticipated above, the factorial structure was developed according to the results of two different statistical analysis: the EFA and the Item–Total Correlation (ITC). Nevertheless, this statistical strategy was not adequate to define a factorial structure, and not one validation study carried out a Confirmatory Factor Analysis (CFA).

According to the systematic review findings and the present critical analysis of the validation studies of the FYI, the measure seems to show some promising characteristics and several limitations. The FYI is an effective tool requiring little administration time that can be applied starting from 11 months of life, both in general populations and those at risk. Therefore, the FYI is a cost-effective measure, appropriate for administration to parents during regular well-child visits as part of pediatric surveillance. Finally, according to the longitudinal research [33], the instrument seems to be an efficient measure for detecting behaviours that deviate from those characterising typical development (and, as such, can be a sign of the risk of ASD).

Nevertheless, the above-mentioned studies have several limitations. First, the cross-cultural generalisability of the FYI was studied on Israeli children. One study [32], involving Italian children, used a retrospective design. It is well known that parental memories may influence the quality of data derived through retrospective methods [37]; thus, further studies are needed to study the cross-cultural generalisability of this measure in a non-American sample. Second, the factorial analysis of the FYI [8] has not confirmed a structure based on the expected eight constructs. It should be noted that the authors did not report the results from the EFA (i.e., factor loadings, percentage of variance explained), and the final structure of the questionnaire was derived from a combination of evidence from the item–total correlations and what they theoretically expected to find. Establishing a psychometrically sound factorial structure of the FYI is not a secondary issue since the calculation of the risk cutoff is based on it. Finally, none of the other studies [31,32,33,34] analysed the factorial structure of the FYI, but rather took for granted what Reznick and colleagues [8] had found. Thus, further demonstrations of the factorial structure are particularly needed.

Therefore, the general aim of the current study was to conduct a screening of the signs of risk of ASD, applying the FYI on an Italian sample (from the general population) undergoing regular well-child visits as part of pediatric surveillance. The study purposes were to (a) examine the cross-cultural generalisability of the FYI, comparing the Italian findings with those of US and Israeli samples (specifically, comparisons of the analyses of socio-demographic variables, response distributions, and cut-offs); (b) demonstrate the construct validity of the FYI; and (c) demonstrate the internal consistency and structural validity of the FYI.

## 2. Materials and Methods

### 2.1. Procedure

The study was carried out in a large urban area in the south of Italy. The Ethical Committee of the Local Public Health Service gave its approval for this research (n° 528/8 March 2017). One hundred fifteen paediatricians of the local public health service received via mail a description of the research project, with a request to collaborate with it. Sixty-four of them (55.6%) participated in the research and received instructions for the recruitment of participants. All families treated by those paediatricians with a child born between February and September of 2016 were invited to participate in the study (*n* = 800). They received a description of the research project and signed informed consent. Data collection was conducted when the parents were at the paediatrician’s office (in a quiet place before the visit); the paediatrician was not present during the administration of the questionnaire.

### 2.2. Measure

Socio-Demographic Variables. The first part of the FYI allows identification of the following information: the child’s gender, date of birth, weight at birth, order of birth, term birth vs. preterm birth, parents’ marital status, their educational level, and their ethnicity. Finally, information was collected as well on who completed the questionnaire (e.g., mother, father, or both). Early identification of signs of risk of ASD. The 63 items of the FYI [8] (Italian translation by Muratori and Narzisi, 2009) allow evaluation of the child’s functioning within two domains: Social–Communication and Sensory–Regulatory Functions. Each domain consists of four constructs. The Social–Communication domain includes the constructs of Social Orienting & Receptive Communication (nine items), Social–Affective Engagement (eight items), Imitation (six items), and Expressive Communication (five items). The Sensory–Regulatory Functions domain includes the constructs of Sensory Processing (six items), Regulatory Patterns (four items), Reactivity (three items), and Repetitive Behaviors (eleven items). According to Reznick and colleagues [8], the final score was calculated through a weighted average of the raw score for each construct and domain. A total score was calculated as an average of the two domains, with higher scores indicating higher risk.

### 2.3. Participants

The convenience sample was composed of 698 returned questionnaires with a response rate of 86.1%. Forty-one questionnaires were excluded from the analyses because they were completed by mothers of children with Down’s Syndrome (*n* = 2) or by mothers of preterm children (i.e., born before the 37th gestation week; *n* = 39). Those children were excluded from the sample since the study purpose was to validate the FYI as a Level 1 screening measure administrable to the general population, that is, children not referred for other developmental concerns. Specifically, the two children with Down’s Syndrome were excluded from the sample because of their genetic disease. Furthermore, the 39 preterm children were excluded since—as in [8] and [32]—they were too immature at 12 months to be evaluated on social and behavioural functioning.

**Figure 1:**
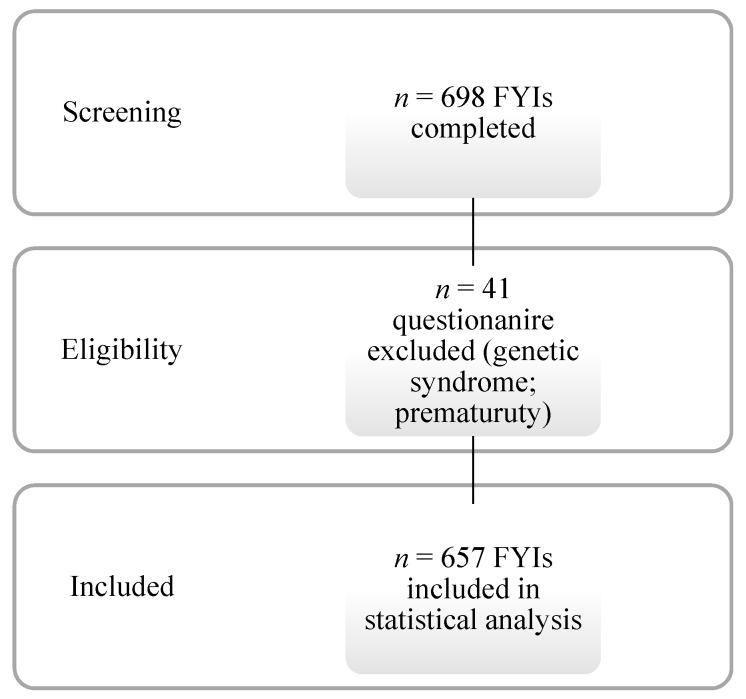
Flowchart of study sample and design.

The final sample was comprised of 657 questionnaires (Figure 1) completed by mothers (69.9%), fathers (5.3%), or both parents together (24.2%) when the children were from 11 to 13 months old (M = 12.4 months; SD = 1 month). Three hundred forty-one of them were boys, 309 were girls. The toddlers’ mean weight at birth was 3.32 kg (SD = 0.51; range 3–4.93 kg); 40.3% of the children were first-born, and 43.7% were second-born or more. The mothers’ mean age was 33.83 (SD = 5.6; range 18–49), and their educational level was low (up to eight years of education) for 26.9% and high (nine or more years of education) for 73.8%. The fathers’ mean age was 37.42 (SD = 6.4; range 19–67), and their mean of the educational level was low (up to eight years of education) for 32.1% and high (nine or more years of education) for 61.3%. The majority of the parents were married (92.8%), whereas 6.4% were single or divorced. The parents were European–White (88.1% of the mothers; 85.7% of the fathers), African (0.6% of the mothers; 1.1% of the fathers), or Asian (1.1% for mothers; 0.3% of the fathers).

### 2.4. Analytic Strategy

Independent sample t-tests were carried out to analyse the differences in the two domains of the FYI (Social–Communication and Sensory–Regulatory Functions), the total score, and the eight constructs based on the socio-demographic variables. When a difference was found as statistically significant, a Cohen’s *d* was reported. To compare the Italian and American (or Israeli) response distributions, a chi-square analysis was run for each item. The null hypothesis (H_0_), that the response distribution of the Italian and American sample (or Israeli) for each item was not different, is what we aimed to demonstrate. Thus, a nonsignificant chi-square is a demonstration that the distributions are comparable. The analyses were conducted in SPSS v.25.

The data were screened to investigate the missing data distribution, normality distribution, and outliers. Exploratory factor analyses (EFA) and confirmatory factor analyses (CFA) through SEM (Structural Equation Modelling) were carried out in Mplus v.8 applying WLSMV because the data were ordinal. Geomin rotation was applied to the EFAs with the Weighted Least Square Mean and Variance (WLSMV) as estimator since the data were ordinal and missing data were also found. The Kaiser–Meyer–Olkin (KMO) statistic was computed on the 60 items of the FYI to evaluate if the data were suitable the data for the factor analysis.

## 3. Results

### 3.1. Preliminary Analysis

Less than 5% of the socio-demographic variables and less than 1.7% for the items of the FYI were missing. Among the latter, those with the highest percentages of missing data were Item 40 (1.7%), Item 5 (1.3%; ‘Does your baby seem to have trouble hearing?’), and Item 16 (1.1%; ‘Is it easy to understand your baby’s facial expressions?’). The ‘Little’s missing completely at random’ test was significant, χ^2^ (3367) = 4008.438; *p* = 0.000; this means that missing data were nonrandomly distributed. For this reason, and given the low percentages, they were not imputed. Comparing our missing patterns with those of the US sample [8], only Item 40 (‘Do your baby’s eyes line up together when looking at an object?’) had a similar percentage of missing data (1.7% in the Italian sample and 2% in the American sample). For all the other items, we had less missing data than the US sample.

### 3.2. Generalisability

Analyses on the socio-demographic variables. The t-tests showed no significant effects by the childbirth order (i.e., first-born = 40.3%; second-born or more = 43.7%) on the FYI domains, the total score, or the eight constructs. With regard to the children’s gender, the t-test showed a significant difference on the Reactivity construct. Boys obtained higher scores than girls. Boys reached higher scores also on the two domains and on the total score. Table 1 shows the results of the t-tests, with means and standard deviations.

Considering the parental socio-demographic variables, the t-tests showed differences for maternal educational level and marital status. Specifically, mothers with a low educational level (up to eight years of education), compared to those with high educational level (nine or more years of education), obtained higher scores in the two FYI domains, the total score, and all constructs, with the exception of Social–Orienting and Receptive Communication (part of the Socio-Communication domain) and Regulatory Patterns (part of the Sensory–Regulatory Functions domain) constructs. Table 2 shows the results of these analyses.

Mothers without a partner showed higher scores (M = 8.03; ds = 7.90) on the Repetitive Behaviors construct (part of the Sensory–Regulatory Functions domain) than mothers with a partner (M = 5.40; ds = 6.48), *t*(44.876) = −2.109, *p* = 0.041.

Comparisons between distributions. We aimed to demonstrate the null hypothesis (H_0_), that the percentage of response distribution for each item for the Italian and American (see Table 3) and Israeli (see Table 4) samples was not different. Indeed, the first column of the Table 3 reports the content of the items, and the second to the fifth columns report the percentages for each response for the two samples.

Comparing the Italian and the American distributions, the χ^2^ values were below the critical values ($\chi_{0.05,3}^{2}$ = 7.815; $\chi_{0.05,2}^{2}$ = 5.991); thus, no differences emerged between the two samples. Similarly, comparing the Italian and the (partially available) Israeli distribution, no difference emerged.

Score Distributions and Cutoffs for ASD Risk. In Table 5, we summarized our scores and those obtained by the other international studies. It was not always possible to compare Italian findings with those of the American and Israeli studies since some data were not available in the papers.

Nevertheless, it is worth noting that the American data range was only theoretical and that the Israeli data range was higher than that in the Italian results. The modal value was 0 both in the American and the Italian data; this confirmed that, in the general population, the majority of FYI scores strived towards the lowest score that indicated typical development. With regard to the mean score, it was possible to compare Italian and Israeli data: the first score was lower than the second. The American mean score was not available.

**Figure 2:**
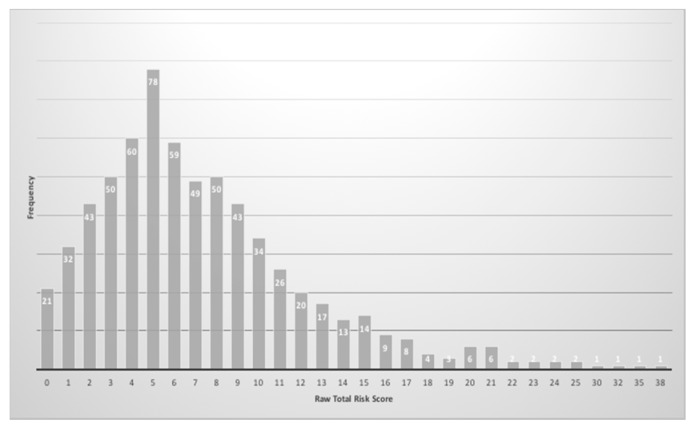
Distribution of First Year Inventory (FYI) raw risk scores in the Italian sample according to the factor structure of Reznick et al. (2007).

Finally, with regards to the cross-cultural risk score comparison, it is worth noting, as few values were reported by the American authors, that the only two values reported in Table 6 were calculated according to Ben-Sasson and Carter’s [31] suggestions. The American and Italian data comparison on risk score on the 95th and 98th percentile showed similar values. Figure 2 shows the distribution of risk score (skewness = 1.53; kurtosis = 4.03) for the Italian sample and the shape inflection corresponding to the score of 17, as found in Reznick and colleagues’ [8] study. Comparing Israeli and Italian data, the Italian raw values corresponding to the 95th and 98th percentile were lower than the Israeli ones.

According to the other two validation studies on a general population [8,31], scores equal or above the 95th percentile could be applied to detect children at risk for ASD. We decided to apply the mean score on the 95th percentile of the total score, which was 8.15; 32 children in our sample met this risk criterion (which corresponds to 4.87% of the sample). A similar result (4.88%) was found by Ben-Sasson and Carter [31] on the Israeli general population. The families with children under the risk condition were invited for a diagnostic assessment with gold standard measures. The evaluation is in progress, and the children have been followed over time.

### 3.3. Construct Validity

To investigate the inter-correlations between the two domains and the eight constructs, Pearson r correlations were carried out. Table 6 reports the correlations between the eight constructs and also the correlations found in Reznick and colleagues’ [8] study as a comparison. As expected, the four constructs of the Social–Communication domain correlated with each other, as did the four constructs of the Sensory–Regulatory Functions domain. Furthermore, results showed that the Expressive Communication construct (part of the Social–Communication domain) did not correlate with all constructs of the Sensory–Regulatory Functions domain; Social–Affective Engagement (part of the Social–Communication domain) did not correlate only with the Sensory Processing and Regulatory Pattern constructs (part of the Sensory–Regulatory Functions domain). The two domains are correlated as well, r = 0.13, *p* = 0.01.

### 3.4. Internal Consistency and Factorial Analyses

The Hayes and Krippendorff’ kalpha for Social–Communication and Sensory–Regulatory Functions domains were 0.91 and 0.88, respectively. These values were higher than those found by Reznick and colleagues [8] and suggested a moderate consistency among items.

**Figure 3:**
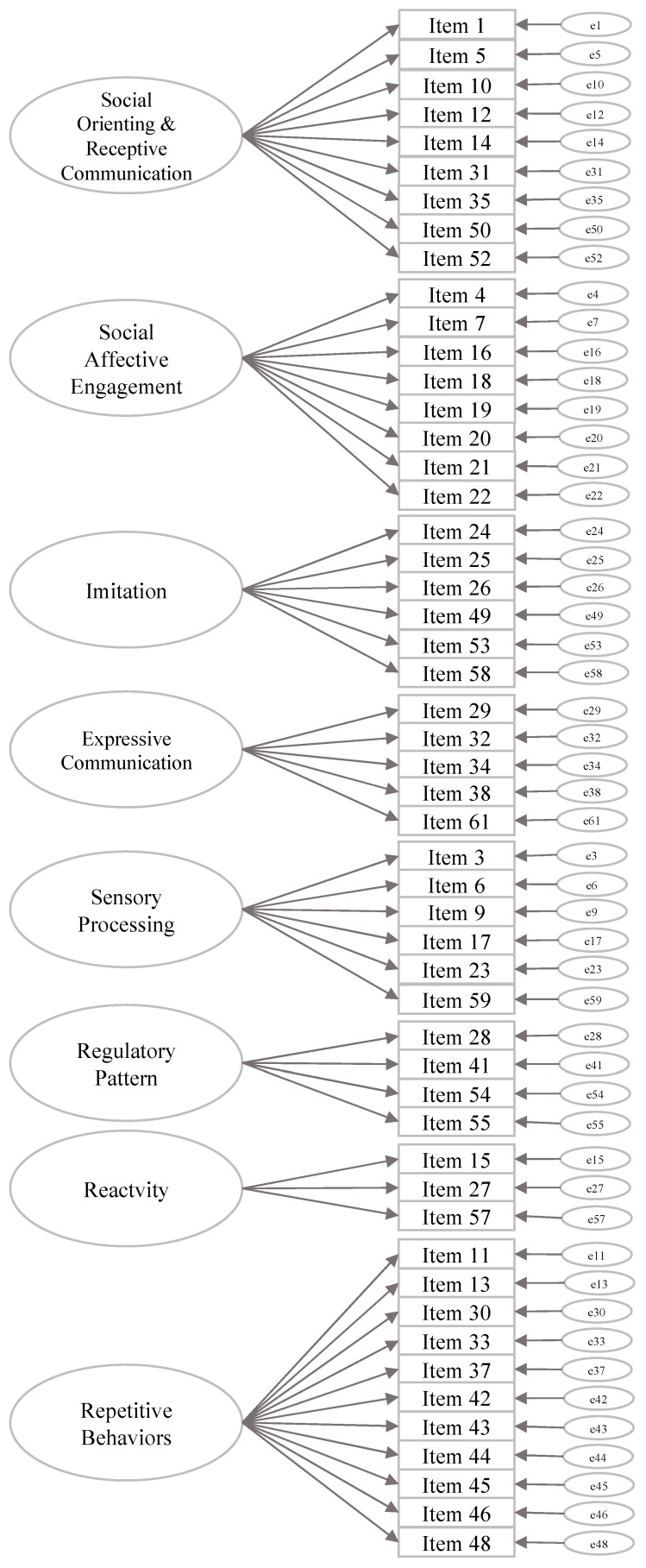
A graphical reproduction of FYI Factor Structure by Reznick et al. (2007).

Exploratory factor analyses (EFA) and confirmatory factor analyses (CFA) through SEM (Structural Equation Modelling) were carried out in Mplus v.8 applying WLSMV because the data were ordinal. Geomin rotation was applied to the EFAs. A first-order CFA was performed on the 52 items of the FYI to test the eight-factor structure corresponding to the constructs hypothesised by Reznick and colleagues [8] (Figure 3). The 10 items that did not load for any factor (see Appendix in Reznick et al.’s paper, [8]) and were not inserted into the analysis. A second-order CFA was tested based on the second-order factorial structure estimating the eight constructs (as first level latent factors) and the two domains (as second-level latent factors). For both CFAs, values of the χ^2^, the CFI (Comparative Fit Index), and the RMSEA (Root Mean Square Error of Approximation) were examined. The two CFAs showed several correlations between items or between constructs with values close or equal to 1, suggesting that the items or the factors should be collapsed.

As both the CFAs failed to estimate acceptable factorial structures, we chose to go back to the EFA. Two factorial structures were tested on the original 61 items: the eight-factor structure, corresponding to the eight constructs, and the two-factor structure, corresponding to the Socio-Communication domain and the Sensory–Regulatory Functions domain. The χ^2^, the CFI, and the RMSEA were examined for both. The items were progressively excluded if the factor loadings loaded for two or more factors or none of them. The comparison between the eight-factor and the two-factor structure showed that the latter was the best fitted. Thus, we performed a further test via CFA.

The first order CFA on the 52 items yielded a moderate–low fit of the data, with a significant χ^2^ (1196) = 2214.53, *p* < 0.001, and CFI = 0.83, RMSEA = 0.036 (LO90% = 0.034, HI90% = 0.038). Similarly, the second order CFA showed moderate-low fit of the data, with a significant χ^2^ (1214) = 2238.99, *p* < 0.001, and CFI = 0.83, RMSEA = 0.036 (LO90% = 0.034, HI90% = 0.038).

The EFA on the 61 items estimating the eight-factor structure was well fitted, χ^2^ (1318) = 1585.34, *p* < 0.001, CFI = 0.96, RMSEA = 0.018 (LO90% = 0.014, HI90% = 0.021). However, 43 items were excluded because the factor loadings loaded for two or more factors and the remaining items loaded for four factors instead of the hypothesised eight, and those four factors did not correspond with the theoretical model hypothesised by Reznick and colleagues [8].

For these reasons, the EFA estimating the two-factor structure was preferred and reached a moderate fit of the data, χ^2^ (1651) = 2940.15, *p* < 0.001, CFI = 0.79, RMSEA = 0.034 (LO90% = 0.032, HI90% = 0.036). Nineteen items (Items 4, 5, 6, 16, 27–29, 31, 32, 39, 41, 49–56) were excluded from the subsequent analysis because the factor loadings loaded for two or more factors. After exclusion of those items, the subsequent fourth EFA reached moderate fit of the data, χ^2^ (739) = 1185.47, *p* < 0.001, CFI = 0.92, RMSEA = 0.03 (LO90% = 0.027, HI90% = 0.033). Items 7, 14, 44, and 48 did not load for any factor and were subsequently deleted. The third EFA reached moderate fit of the data, χ^2^ (593) = 996.58, *p* < 0.001, CFI = 0.92, RMSEA = 0.03 (LO90% = 0.029, HI90% = 0.036), and again, Items 11 and 57 did not load for any factor and were subsequently deleted. A final EFA was carried out with the remaining items (Factor 1: *n* = 15 items; Factor 2: *n* = 16 items), again showing moderate fit of the data, χ^2^ (526) = 921.79, *p* < 0.001, CFI = 0.92, RMSEA = 0.034 (LO90% = 0.030, HI90% = 0.037). Table 7 shows the final EFA solution. Factor 1 contains items corresponding to the Social–Communication Domain, Factor 2 to the Sensory–Regulatory Functions Domain, so all the items loaded for the expected factor.

**Figure 4:**
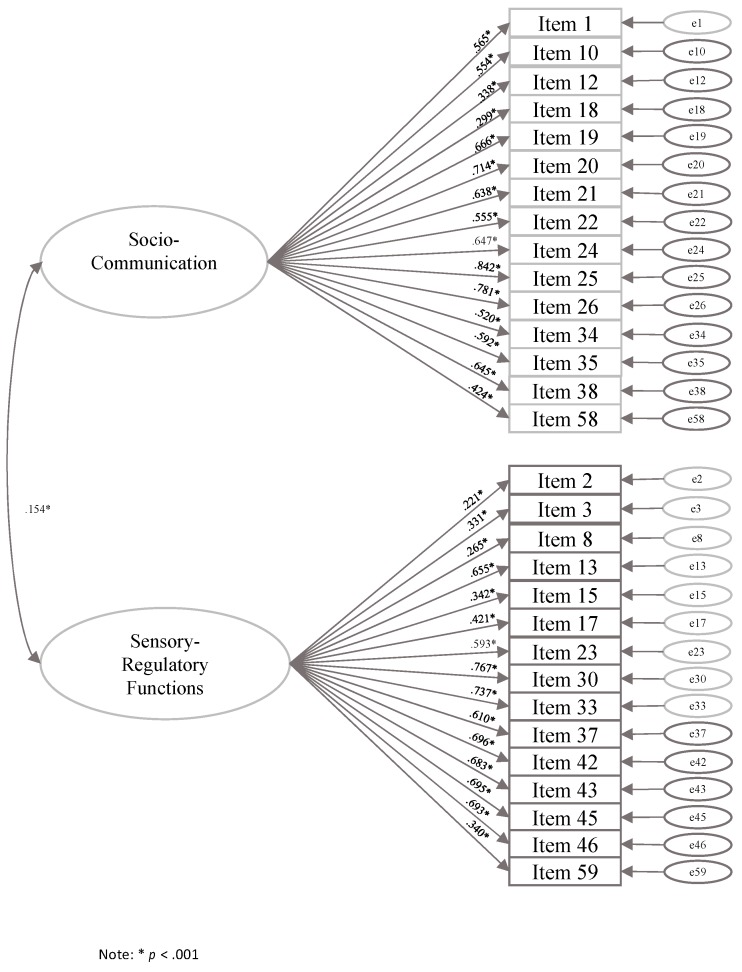
FYI Structure according to Confirmatory Factor Analysis (CFA) run in this study.

The final EFA structure was tested via CFA. The two-factor structure showed moderate fit of the data, χ^2^ (433) = 672.72, *p* < 0.001, CFI = 0.95, RMSEA = 0.029 (LO90% = 0.026, HI90% = 0.033). Item 9 had low factor loading with the factor and was subsequently deleted. The two factors were weakly correlated, r = 0.15, *p* = 0.045. The final CFA was carried out showing good fit of the data, χ^2^ (404) = 617.699, *p* < 0.0001, CFI = 0.95, RMSEA = 0.028 (LO90% = 0.024, HI90% = 0.033). Figure 4 shows the factor structure obtained by the CFA.

After those analyses, we re-examined our data according to the new factorial structure. The total score ranged from 0 to 18.39, with a mean of 3.27 (SD = 3.04), a median of 2.17, and a distribution shaped as a chi-square (skewness = 1.49; kurtosis = 2.86). The t-tests showed a significant difference by children’s gender, *t*(648) = 2.062, *p* = 0.040, with boys reaching a higher total score (M = 3.48; ds = 3.06) than the girls (M = 2.99; ds = 2.95). There were no significant differences by childbirth order or by parents’ marital status. In contrast, the t-tests showed significant differences by educational level on the Sensory–Regulatory Functions domain, *t*(208.185) = 3.537, *p* < 0.0001 and on the total score, *t*(206.433) = 3.755, *p* < 0.0001. Specifically, mothers with a low educational level showed higher scores (Sensory–Regulatory Functions domain: M = 6.70; ds = 5.98; total score: M = 4.18; ds = 3.51) than mothers with a high educational level (Sensory–Regulatory Functions domain: M = 4.79; ds = 4.87; total score: M = 2.99; ds = 2.82). Finally, the risk cutoff on the 95th percentile of the total score corresponded to a score of 9.14.

## 4. Discussion

The main purpose of this study was to conduct an early screening of the signs of risk of ASD, applying the FYI as part of pediatric surveillance on an Italian sample from the general population. We examined the cross-cultural generalisability of the screening measure, comparing the Italian scores with those of the two validation studies conducted on a general population [8,31]. The other two aims of the research were to test the construct validity of the FYI and to demonstrate its internal consistency and structural validity.

The combination of all the results mentioned represents a demonstration of the generalisability and stability of the measure across cultures. First of all, we considered the role played by the socio-demographic variables and compared the present findings with those found with the American and Israeli samples. Significant differences were found by children’s gender, with boys showing higher scores than girls for the Reactivity construct (part of the Sensory–Regulatory Functions domain).

Considering the parental variables, it is worth noting that in the other two validation studies on the general population [8,31], among the socio-demographic variables considered, the authors examined whether maternal ethnicity influenced the scores of the FYI (Reznick et al., 200). Those differences were not tested on the Italian sample, because all the parents were European–White. Reznick and colleagues [8] and Ben-Sasson and Carter [31] also considered the educational level and marital status of the mothers.

Similarities between the Italian and American and Israel samples were also found for the maternal educational level and marital status. As Reznick and colleagues [8] and Ben-Sasson and Carter [31] found, a low educational level was associated with higher FYI scores compared to a high educational level. One possible explanation is that mothers with a low educational level may interpret several atypical behaviours as common because they misunderstood the meaning of the item [31]. In particular, the items of the Sensory–Regulatory Functions domain describe atypical behaviours, as they would be ‘positive’ (i.e., presence of a behaviour) instead of ‘negative’ (i.e., absence of a developmentally expected behaviour). For example, the item, ‘Is your baby content to play alone for an hour or more at a time?’ can be misleading because the mothers may interpret as positive the fact that child plays quietly alone for long periods (i.e., presence of a behaviour).

Moreover, we found that single mothers reported higher FYI scores on the Repetitive Behaviors construct (part of the Sensory–Regulatory Functions domain) than did married mothers. Ben-Sasson and Carter [31] found a similar result for the Sensory–Regulatory Functions domain. The explanations of these results may be twofold. First, the single mothers did not have a partner with whom they could discuss concerns about the child’s development; thus, they could interpret the child’s Sensory–Regulatory behaviours as atypically. Second, the child’s self-regulation process may be affected by the absence of the father [38].

As a further demonstration of the cross-cultural generalisability of the FYI, we found similar patterns of response for each item, meaning that there were no differences across cultures in the way in which parents of children from 11 to 13 months of age replied to the questions. This result highlighted that targeted behaviours evaluated by the FYI were identifiable in a similar manner across different cultures. Thus, this property allows the detection of typical and atypical behaviours that appear to be cross-culturally invariant.

Finally, the Italian results were similar to the American findings for the total risk score calculated on the 95th and 98th percentile, and both were lower than the risk scores calculated on the Israeli sample. As Ben-Sasson and Carter [31] suggested, this could be due to the dysregulation [39] and the stress [40] endured by Israeli children growing-up in a stressed society faced with trauma and terror daily.

Nevertheless, the percentage (32%) of children detected at risk (with a total score equal or above the 95th percentile) in the Italian and the Israeli samples was similar (these data were not available in Reznick and colleagues’ [8] study).

The second aim examined the FYI construct validity. The positive and significant correlations between the two domains of the instrument (Social–Communication and Sensory–Regulatory Functions) and between constructs highlighted a good construct validity of the measure, as found by Reznick and colleagues [8].

Since no previous studies on the FYI have validated its factorial structure, the purpose of the present study was to give insight on this property. In this vein, the Confirmatory Factor Analysis is a crucial and strategical analysis demonstrating the structural validity of a measure. Therefore, we firstly carried out a CFA on the theoretical structure hypothesized by Reznick and colleagues [8]. Our analyses did not confirm the structure of the scale organised on the eight hypothesised constructs. It should be noticed that Reznick and colleagues [8] also struggled to find a stable factorial solution for their data and decided to shape the final eight constructs through the item–total correlations and the expected thematic content of the items. As the second step in our study, two second-order latent factors, corresponding to the two main domains of the FYI, were estimated through CFA. Even in this case, the results did not support the hypothesised structure. Therefore, we decided to explore the structure of our data with a set of five nested EFAs in which several items were found as critical, because of loading more than one factor or because of not showing the expected factor loading (i.e., > 0.30), and deleted step by step. The final explorative factorial structure comprehended 30 items, which are coherently distributed in the Social Communication and Sensory–Regulatory Functions domains. A CFA confirmed this structure and allowed the estimation of a short version of the FYI, which was suggested by Turner-Brown and colleagues [34] as one point to be developed by future research after their study. The short version of the questionnaire makes its administration easier and faster and allows applying the questionnaire during systematic screening evaluations on the general population.

The short version of the FYI evaluated the two main core areas of risk for ASD, in which the main symptoms are included, as suggested by Reznick and colleagues [8] and the DSM-5 [1]. Most of the items of the short version assess the social and communicative deficit (Factor 1), focusing on the evaluation of receptive communication and social engagement. The others evaluate the first factor focussing on child’s imitative capacity, and his/her expressive communication. Furthermore, the second factor estimated in the short version (Sensory–Regulatory Functions domain) evaluates the presence of repetitive behaviours and the hypo- or hypersensitivity of the child to sensory stimuli. The evidence on the FYI short version highlighted the expected results considering both the parental and the children’s socio–demo variables, as found in the other validation studies [8,32] who applied the full version of FYI.

The total score calculated on the final structure of the scale showed significant difference by gender, with boys reaching higher total scores compared to the girls, confirming the American and Israeli findings and the gender ratio of ASD (4:1) [1]. Even with the total score calculated on the short version (FYI-30), low parental educational level was associated with higher total score compared to the opposite condition, whereas marital status was not significant. Therefore, the estimated short version seems to represent the two core symptoms of the ASD and, at the same time, maintains the impact of the socio-demographic variables on the total score as found by previous research.

## 5. Limitation

The main limitation of the present study is the cross-sectional design. Longitudinal studies on the general population are required to demonstrate the accuracy of the FYI, its PPV (i.e., positive predictive value) and NPV (i.e., negative predictive value), and ability to detect signs of risk of ASD. Future studies, starting from our results on the FYI short version, should consider the diagnostic outcome evaluation, through gold-standard measures, and the convergent validity. Specifically, the evaluation should be focused on the severity of the autistic traits, the global child development, and characteristics of attention-selectivity processes [41]. A prospective study is currently ongoing with a longitudinal evaluation of children considered to be at risk at 11–13 months of life and evaluated one and two years later. Furthermore, other studies should further demonstrate the short version structure of the FYI developed in this study. The second limitation is related to the relatively low response rate of the professionals in our study, although it is similar to what was found by others [8,32]. It should be noticed that the low response rate of the professionals did not correspond to a similar low parental response rate. Indeed, when the paediatrician participation was obtained, on their side, parents easily agreed to be participants. It is highly likely that parental participation depends on the quality of their relationship with the paediatrician, as found by others [42,43]. This also means that a way to establish a continuous screening for children’s mental health and speed up early diagnosis and intervention is increasing health professionals’ awareness of that aspect.

## 6. Conclusions and Implication 

According to our results, the FYI is a valid and reliable screening tool for Italian children. Results for the current study stimulate further research in the field of cross-cultural validity and generalisability of the FYI and other measures for the early identification of signs of risk of ASD.

Our findings highlighted some positive features of the FYI and, at the same time, several others that should be further developed. On the one hand, the analyses have shown the cross-cultural stability and generalisability of the FYI as well as its construct validity. Therefore, the FYI is a reliable tool that may be administered in another cultural context from the American and Israeli ones.

On the other hand, modest demonstrations of internal consistency were found, as also confirmed by the factorial analyses. As for the latter, the hypothesised structure (see [8] for details) did not receive appropriate support, showing poor fit of the data with several correlations between items with values close or equal to 1. The alternative analyses carried out revealed a structure organised on the two main core symptoms of ASD, also identified by Reznick and colleagues [8] in their original version of the FYI, based on a short version of the questionnaire. Our analyses demonstrated that the factorial validity of the FYI requires further demonstration. This notwithstanding, the short version of the FYI may lead to a cost-effective and easy-to-administer instrument to be used by paediatricians during their pediatric surveillance on the general population. The early detection of atypical developmental trajectories may support medical decision-making on further steps for referral of the child to an early diagnostic assessment (which may enable early intervention when needed; [43,44,45,46,47,48].

## Figures and Tables

**Table 1:** Independent-sample t-test by gender on the FYI domains, total score, and constructs.

	Males M(ds)	Females M(ds)	*t*	Cohen’s *d*
**Social–Communication domain**	2.83 (3.08)	2.64 (3.03)	*t*(648) = 0.766	-
Social orienting & receptive communication	1.13 (2.69)	1.26 (2.84)	*t*(648) = −0.582	-
Social-affective engagement	2.02 (3.70)	2.23 (4.01)	*t*(648) = −0.691	-
Imitation	1.44 (3.79)	1.29 (4.25)	*t*(648) = 0.467	-
Expressive communication	6.71 (7.79)	5.79 (7.17)	*t*(648) = 1.567	-
**Sensory–Regulatory Functions domain**	4.07 (4.01)	3.49 (3.65)	*t*(648) = 1.921	-
Sensory processing	3.71 (5.14)	3.72 (5.32)	*t*(648) = −0.029	-
Regulatory patterns	4.54 (9.35)	3.72 (7.8)	*t*(643.626) = 1.224	-
Reactivity	2.08 (5.32)	1.26 (4.42)	*t*(643.169) = 2.145 *	0.17
Repetitive behaviors	5.94 (7.02)	5.25 (6.41)	*t*(648) = 1.303	-
**Total score**	3.44 (2.68)	3.06 (2.52)	*t*(648) = 1.867	-

Note: * *p* < 0.05.

**Table 2:** Independent-sample t-tests by maternal educational level on the FYI domains, total score, and constructs.

	Low Educational Level M(ds)	High Educational Level M(ds)	*t*	Cohen’s *d*
**Social–Communication domain**	3.45 (3.76)	2.54 (2.80)	*t*(197.576) = 2.736 *	0.27
Social orienting & receptive communication	1.04 (2.54)	1.22 (2.82)	*t*(630) = −0.695 *	0.07
Social–affective engagement	2.98 (4.80)	1.82 (3.46)	*t*(194.269) = 2.719 *	0.27
Imitation	1.90 (5.23)	1.26 (3.60)	*t*(189.821) = 1.378	-
Expressive communication	7.90 (8.37)	5.84 (7.19)	*t*(215.355) = 2.692 *	0.26
**Sensory–Regulatory Functions domain**	4.65 (4.15)	3.59 (3.81)	*t*(630) = 2.895 *	0.06
Sensory processing	4.90 (6.18)	3.45 (4.93)	*t*(205.617) = 2.594 *	0.26
Regulatory patterns	4.03 (7.93)	4.37 (9.20)	*t*(630) = −0.406	-
Reactivity	2.52 (5.59)	1.44 (4.59)	*t*(208.993) = 2.121 *	0.21
Repetitive behaviors	7.16 (7.51)	5.10 (6.33)	*t*(212.843) = 3.028 *	0.30
**Total score**	4.05 (3.03)	3.06 (2.45)	*t*(207.011) = 3.615 *	0.36

Note: * *p* < 0.05.

**Table 3:** Chi-square comparison between American (AS; Reznick et al., 2007) and Italian (ItS) sample distribution (%).

	Never		Seldom		Sometimes		Often	
	ItS	AS	ItS	AS	ItS	AS	ItS	AS
1. Does your baby turn to look at you when you call your baby’s name?	0	<1	0.3	1	2.3	8	97.4	91
2. Does your baby seem bothered by loud sounds?	21.2	8	25.3	39	41.6	46	11.7	7
3. Does your baby seem overly sensitive to your touch (for example, fuss or pull away when you touch him or her)?	71.2	64	19.4	31	6.2	5	2.4	<1
4. During familiar games like ‘‘I’m gonna get you,’’ does your baby get excited because he or she knows what will happen next?	1.4	<1	0.6	<1	7	8	90.7	92
5. Does your baby seem to have trouble hearing?	97	94	1.2	5	0.2	1	0.5	<1
6. When you and your baby are facing each other, does your baby turn his or her eyes to avoid looking at you?	81.7	53	11.9	30	4.7	15	1.1	2
7. In new or strange situations, does your baby look at your face for comfort?	3.3	1	7.2	6	28.6	40	60.4	53
8. Does your baby ignore loud or startling sounds?	65.3	34	20.5	42	10.7	21	2	3
9. Does your baby spit out certain textures of foods, such as lumpy or chunky pieces?	25.3	11	18.4	25	35.3	48	19.6	16
10. When you point to something interesting, does your baby turn to look at it?	0.5	1	1.4	4	13.2	39	83.9	56
11. Is your baby content to play alone for an hour or more at a time?	35.6	27	28.2	29	22.2	31	13.1	13
12. Does your baby look at people when they begin talking, even when they are not talking directly to your baby?	0.3	<1	1.4	3	15.2	44	83	53
13. Does your baby rock his or her body back and forth over and over?	61	54	14.8	24	16.7	15	7	7
14. Does your baby look up from playing with a favorite toy if you show him or her a different toy?	1.7	<1	3.8	2	31.1	39	62.7	59
15. Does your baby get upset when you need to switch your baby from one activity to another one?	34.1	7	28.5	35	30	53	7	5
16. Is it easy to understand your baby’s facial expressions?	0.8	<1	0.9	1	6.1	14	90.7	85
17. Does your baby forcefully press his or her face, head, or body against people or furniture?	79.6	38	10	27	7.6	24	2.1	11
18. Does your baby smile while looking at you?	0.2	<1	0.2	<1	5.2	9	94.2	91
19. Does your baby try to get your attention to show you something interesting?	1.4	7	3.5	16	26.6	40	67.9	37
20. Does your baby try to get your attention to play games like peek-a-boo?	3.8	5	5	15	25	41	65.4	39
21. Does your baby try to get your attention to obtain a favorite toy or food?	1.4	2	2.3	9	14.6	32	81.1	57
22. Does your baby try to get your attention to play physical games, like swinging, tickling, or being tossed in the air?	4.6	10	9.1	23	33.9	40	51.9	26
23. When your baby is awake and you pick him or her up, does your baby’s body feel loose or floppy?	87.4	81	7	14	3.3	4	1.4	1
24. Does your baby copy or imitate you when you make sounds or noises with your mouth?	0.9	1	2.6	4	22.7	32	73.5	63
25. Does your baby copy or imitate your actions, like sticking out your tongue, clapping your hands, or shaking your head?	0.9	<1	1.5	2	9.9	23	87.5	75
26. Does your baby copy or imitate you when you do something with a toy or object, like shaking a rattle or banging a spoon on the table?	0.8	<1	1.1	1	9.7	22	88.3	77
27. Is it difficult to calm your baby once he or she becomes upset?	33.9	20	41.7	62	19.9	17	4.1	1
28. Are your baby’s sleeping and waking patterns regular from day to day?	1.7	1	6.1	4	11.8	20	80.2	75
29. Does your baby try to get your attention by making sounds and looking at you at the same time?	3.7	1	6.4	4	26.5	30	62.7	65
30. Does your baby get stuck doing a simple activity over and over?	79.3	36	14.6	45	4	16	1.1	3
31. Does your baby seem interested in other babies his or her age?	0.5	<1	0.8	5	9.5	28	89	67
32. Does your baby babble by putting sounds together, such as ‘ba-ba’, ‘ga-ga-ga’, or ‘ba-dee’?	8.1	<1	3.2	1	11.7	8	76.6	91
33. Does your baby enjoy staring at a bright light for long periods of time?	62.56	49	22.1	32	11.7	15	2.9	4
34. Does your baby use gestures such as raising arms to be picked up, shaking head, or waving bye-bye?	0.2	<1	0.3	3	3.7	12	95.9	85
35. When you say ‘‘Where’s (a familiar person or object)?’’ without pointing or showing, will your baby look at the person or object named?	0.6	4	2	10	13.7	35	83.3	51
36. Does your baby use the first finger and tip of the thumb to pick up a very small object like a raisin or a Cheerio?	1.4	<1	1.9	1	5.9	5	90.4	94
37. Does your baby seem to get stuck on playing with a part of a toy (such as an eyeball, label, wheel or tag), instead of the whole toy?	16.1	14	18.1	32	34.1	39	31.4	15
38. Does your baby communicate with you by using his or her finger to point at objects or pictures?	5.6	12	5.8	18	19.2	24	69.4	46
39. Do you get the feeling that your baby plays or communicates with you less now than in the past?	87.8	80	4.4	14	1.7	5	5.3	1
40. Do your baby’s eyes line up together when looking at an object?	5.8	1	1.5	1	4.3	3	85.8	95
41. Are your baby’s feeding patterns regular from day to day?	1.2	1	1.1	2	7.3	19	90	78
42. Does your baby enjoy rubbing or scratching toys or objects for long periods of time?	40.6	49	21	34	22.8	13	14.9	4
43. Does your baby seem to get his or her body stuck in a position or posture that is hard to move out of?	77.9	70	13.7	23	6.2	6	1.2	1
44. Does your baby enjoy making objects spin over and over in the same way?	43.2	32	21.4	33	26.3	27	8.7	8
45. While lying down, does your baby enjoy kicking his or her feet over and over for long periods of time?	32.1	42	18.1	33	30.1	19	19.2	6
46. Does your baby stare at his or her fingers while wiggling them in front of his or her eyes?	47.8	32	17.4	35	24.4	27	10	6
47. Which of the following best describes your baby’s typical play with a favorite toy?	10.4	12	30.6	55	58.3	33		
48. Which of the following describes your baby’s interest in toys on a typical day?	4.7	3	23.4	27	71.2	70		
49. When you introduce your baby to a new game (peek-a-boo, so-big, patty-cake, etc.) how does your baby respond?	86.6	29	11.9	63	0.9	6	0.2	2
50. What do you typically have to do to get your baby to look up from playing with a favorite toy?	68.2	43	25.4	54	5.9	3		
51. What is your baby’s usual reaction to somewhat painful experiences, like bumping his or her head?	2.7	4	89.2	93	7.5	3		
52. What do you typically have to do to get your baby to turn towards you?	88.9	71	9.3	25	1.5	4		
53. What do you typically have to do to get your baby to smile or laugh at you?	92.1	92	6.5	8	0.9	<1		
54. On a typical night, how many hours does your baby sleep?	4.9	13	36.7	71	46.9	14	11.1	2
55. On a typical night, how many times does your baby wake up?	27.5	51	55.1	43	16.7	6		
56. Which of the following best describes your baby’s skill level?	19.8	48	44.9	44	29.5	6	5.2	2
57. Which of the following best describes your baby’s typical day?	76.3	28	21.2	59	1.7	11	0.2	2
58. If you start a game by copying or imitating a sound your baby makes, what does your baby typically do?	0.6	<1	7	11	26.9	35	64.4	54
59. When your baby is awake and not eating, does your baby keep a toy or object in his or her mouth?	22.1	29	37.4	50	30.6	17	9.4	4
60. Which of the following best describes the way your baby coordinates his or her eyes and hands while playing with a toy?	89.8	81	7.3	19	1.5	<1	0.9	<1

Note: IS = Italian Sample; AS = American Sample. The bold line identifies the items with three or four multiple-choice answers.

**Table 4:** Chi-square comparison between Italian (ItS) and Israeli (IS; Ben-Sasson and Carter, 2012) sample response distribution (%).

	Never		Seldom		Sometimes		Often	
	ItS	ISS	ItS	ISS	ItS	ISS	ItS	ISS
3. Does your baby seem overly sensitive to your touch (for example, fuss or pull away when you touch him or her)?	71.2	83	19.4	14	6.2	2.1	2.4	1.3
6. When you and your baby are facing each other, does your baby turn his or her eyes to avoid looking at you?	81.7	70	11.9	21	4.7	7	1.1	1
9. Does your baby spit out certain textures of foods, such as lumpy or chunky pieces?	25.3	26	18.4	38	35.3	30	19.6	7
13. Does your baby rock his or her body back and forth over and over?	61	39	14.8	25	16.7	31	7	10
17. Does your baby forcefully press his or her face, head, or body against people or furniture?	79.6	59	10	23	7.6	15	2.1	3
23. When your baby is awake and you pick him or her up, does your baby’s body feel loose or floppy?	87.4	26	7	16	3.3	28	1.4	30
30. Does your baby get stuck doing a simple activity over and over?	79.3	25	14.6	40	4	31	1.1	4
35. When you say “Where’s (a familiar person or object)?” without pointing or showing, will your baby look at the person or object named?	0.6	11	2	14	13.7	39	83.3	36
37. Does your baby seem to get stuck on playing with a part of a toy (such as an eyeball, label, wheel or tag), instead of the whole toy?	16.1	9	18.1	20	34.1	37	31.4	34
43. Does your baby seem to get his or her body stuck in a position or posture that is hard to move out of?	77.9	53	13.7	38	6.2	8	1.2	2
48. Which of the following describes your baby’s interest in toys on a typical day?	4.7	5	23.4	41	71.2	55		
55. On a typical night, how many times does your baby wake up?	27.5	20	55.1	61	16.7	19		
56. Which of the following best describes your baby’s skill level?	19.8	14	44.9	54	29.5	26	5.2	6
58. If you start a game by copying or imitating a sound your baby makes, what does your baby typically do?	0.6	0.4	7	19	26.9	51	64.4	30

Note: ITS = Italian Sample; IS = Israeli Sample. The bold line identifies the items with three or four multiple-choice answers.

**Table 5:** Comparison between the American, Israeli and Italian cutoffs.

	Reznick et al., (2007) *n* = 1300	Ben-Sasson and Carter, (2012) *n* = 471	Italian Sample *n* = 657
**Range**	0–50 (theoretical range)	0–33.88	0–20.32
**Modal score**	0		0
**Median score**	5.75	9.13	2.74
**Mean score**	-	10.40 (*sd* = 6.38)	3.29 (*sd* = 2.74)
**Total risk score (≥95th percentile)**	17.75 *	22.55	17
**Total risk mean score (≥95th percentile)**	-	-	8.15
**Social–Communication domain score (95th percentile)**	-	27.85	7
**Sensory–Regulatory Functions domain (95th percentile)**	-	26.95	10
**Total risk score (98th percentile)**	22.62 **	28.14	21
**Children at risk on 95th percentile**	-	4.88%	4.87%

* Ben-Sasson and Carter reported that this value was from a personal communication by Reznick. ** This value was not reported in Reznick and colleagues (2007), it was calculated according to Ben-Sasson and Carter (2012). “-” Means the values were not reported in the paper.

**Table 6:** Correlations among the FYI 8 constructs on the Italian sample. Between parentheses, the results of the correlations yielded in the American sample by Reznick and colleagues (2007).

FYI Construct	Social–Communication Domain			Sensory–Regulatory Functions Domain			
	1	2	3	4	5	6	7
Social Orienting a Receptive Communication	0.16 *** (0.42 **)	0.32 *** (0.38 **)	0.12 *** (0.42 **)	0.07 (0.19 **)	0.08 * (0.10 **)	0.10 * (0.13 **)	0.13 ** (0.12 **)
Social-Affective Engagement (1)		0.28 *** (0.33 **)	0.30 *** (0.49 **)	−0.01 (0.03)	0.04 (0.04)	0.08 * (−0.01)	0.11 ** (0.04)
Imitation (2)			0.20 *** (0.35 **)	0.07 (0.12 **)	0.09 * (0.03)	0.13 *** (0.10 **)	0.09 * (0.02)
Expressive Communication (3)				−0.05 (0.07)	0.05 (0.03)	0.06 (0.03)	−0.01 (0.04)
Sensory Processing (4)					0.14 *** (0.18 **)	0.13 ** (0.30 **)	0.34 *** (0.38 **)
Regulatory Pattern (5)						0.08 * (0.15 **)	0.06 (0.11 **)
Reactivity (6)							0.14 *** (0.10 **)
Repetitive Behavior (7)							

Note: * *p* < 0.05; ** *p* < 0.01; *** *p* < 0.001; df: 655.

**Table 7:** Exploratory Factor Analysis (EFA) results (standard errors between parentheses).

	1	2
FYI_1	**0.544 (0.091)**	−0.196
FYI_10	**0.550 (0.051)**	−0.092
FYI_12	**0.345 (0.055)**	−0.025
FYI_18	**0.304 (0.082)**	−0.045
FYI_19	**0.655 (0.039)**	−0.159
FYI_20	**0.708 (0.036)**	−0.131
FYI_21	**0.625 (0.047)**	−0.168
FYI_22	**0.570 (0.038)**	−0.005
FYI_24	**0.653 (0.037)**	−0.046
FYI_25	**0.851 (0.031)**	−0.003
FYI_26	**0.787 (0.034)**	−0.004
FYI_34	**0.522 (0.080)**	−0.047
FYI_35	**0.578 (0.049)**	−0.160
FYI_38	**0.642 (0.036)**	−0.114
FYI_58	**0.425 (0.047)**	−0.040
FYI_2	0.017	**0.239 (0.044)**
FYI_3	−0.037	**0.335 (0.051)**
FYI_8	−0.061	**0.258 (0.048)**
FYI_9	0.054	**0.198 (0.046)**
FYI_13	−0.106	**0.651 (0.035)**
FYI_15	−0.087	**0.337 (0.041)**
FYI_17	0.003	**0.434 (0.052)**
FYI_23	−0.149	**0.583 (0.059)**
FYI_30	−0.049	**0.774 (0.036)**
FYI_33	−0.158	**0.729 (0.029)**
FYI_37	−0.112	**0.607 (0.032)**
FYI_42	−0.048	**0.701 (0.027)**
FYI_43	−0.080	**0.682 (0.039)**
FYI_45	−0.072	**0.695 (0.028)**
FYI_46	−0.075	**0.693 (0.028)**
FYI_59	−0.034	**0.343 (0.040)**
